# East Suffolk and Ipswich Hospital

**Published:** 1913-08-02

**Authors:** 


### East Suffolk and Ipswich Hospital.

This institution held its seventy-seventh annual meeting at the Town Hall, Ipswich, on July 19; the veteran President, Dr. J. H. Bartlet, presided. The report from the board of management contained some references of more than passing interest. The annual subscriptions received were in excess of any previous year, while the Employés' Organisation only fell short of the record by £59. During the year a new dental department was installed, and an addition was made to the out-patient department, including rooms for *x*-ray work, electric baths, a casualty theatre, and surgeries. The difficulty of finding residents resulted in the appointment of a lady doctor to the vacant post of house physician. The increased price of all commodities, coupled with an increase in the number of in-patients, advanced the ordinary expenditure to such an extent that a deficit of £575 accrued on the year's working. The board pointed to the necessity for balconies to the main wards and for a convalescent home in connection with the hospital, but await the beneficence of individuals rather than sell securities at the present moment. The salary of the secretary, Mr. A. Griffiths, was increased, and a cheque was awarded to him as an honorarium for special services. During the three and a half years that Mr. Griffiths has held the appointment at Ipswich he has changed the system of accounts of that institution to that of the Uniform System. He has also written some articles on hospital accountancy which have appeared in this Journal. It is not always that capable officers obtain recognition in so tangible a form, and we congratulate Mr. Griffiths.

